# Preoperative Systemic Immune–Inflammation Index as an Independent Predictor of Postoperative Wound Infection in Diabetic CABG Patients

**DOI:** 10.3390/jcdd13040164

**Published:** 2026-04-10

**Authors:** Hakan Öntaş, Asiye Aslı Gözüaçık Rüzgar

**Affiliations:** 1 Department of Cardiovascular Surgery, Balikesir University, Balıkesir 10145, Türkiye; 2 Department of Cardiovascular Surgery, Balikesir Atatürk City Hospital, Balıkesir 10100, Türkiye; drasli30@gmail.com

**Keywords:** Coronary Artery Bypass, Diabetes Mellitus, wound infection, inflammation mediators, postoperative complications

## Abstract

**Background:** This study evaluated the independent predictive value of preoperative Systemic Immune–Inflammation Index (SII) for postoperative wound infection (WI) in diabetic patients undergoing isolated Coronary Artery Bypass Grafting (CABG). **Methods:** A retrospective cohort of 300 diabetic patients (2024–2025) was analyzed. The primary outcome was 30-day postoperative WI. Preoperative SII was calculated from blood counts within 24 h before surgery. Multivariable logistic regression was performed using both a primary model (adjusting for age, BMI, and comorbidities) and an extended model including glycemic control (HbA1c), smoking status, operative duration, and transfusion requirements. Model discrimination was evaluated via Area Under the ROC Curve (AUC). Statistical power and sensitivity analyses were conducted to ensure the robustness of the findings. **Results:** WI occurred in 7% (*n* = 21). Preoperative SII was significantly lower in the WI group (958.48 ± 493.49 vs. 1293.56 ± 758.15, *p* = 0.047). SII remained an independent predictor in the adjusted model (Adjusted OR per 100-unit increase: 0.93; 95% CI: 0.86–1.00; *p* = 0.048). ROC analysis confirmed an inverse predictive pattern (AUC: 0.374, 95% CI: 0.312–0.436). Comparative analysis showed that SII provided superior additional insight compared to NLR and PLR in this population. **Conclusions:** Preoperative SII is an independent predictor for WI in diabetic CABG patients. However, given the modest discriminative performance (AUC: 0.374), it should be integrated into a broader clinical risk assessment. Contrary to conventional expectations, lower SII values indicated increased susceptibility, suggesting that immune exhaustion rather than hyperinflammation may drive infectious risk in diabetic patients.

## 1. Introduction

Coronary Artery Bypass Grafting (CABG) is widely recognized as the gold-standard revascularization strategy for patients with multivessel coronary artery disease or complex coronary lesions, particularly when the extent of disease limits the effectiveness of percutaneous coronary interventions [1]. Despite its established benefits in long-term survival and symptomatic improvement, postoperative outcomes vary substantially depending on patient-specific comorbidities. Among these, Diabetes Mellitus (DM) stands out as one of the most influential determinants of surgical risk. DM is associated with accelerated atherosclerotic progression, endothelial dysfunction, impaired microvascular blood flow, oxidative stress, and chronic low-grade inflammation, which together contribute to adverse perioperative and postoperative outcomes [2]. Large-scale cohort studies confirm that DM is linked to increased long-term mortality, recurrent myocardial ischemia, and higher rates of heart failure following CABG, further underscoring its significance as a major contributor to surgical morbidity [2,3,4,5].

The chronic inflammatory and immune-dysregulated milieu characteristic of diabetes creates a postoperative environment uniquely vulnerable to complications. Hyperglycemia impairs neutrophil chemotaxis, reduces phagocytic activity, disrupts lymphocyte proliferation, and delays fibroblast-mediated collagen deposition—key processes necessary for effective tissue repair. In addition, the accumulation of advanced glycation end-products (AGEs), oxidative stress, and microangiopathy limits oxygen delivery to surgical tissues, further amplifying susceptibility to infection [6]. Recent mechanistic studies have demonstrated that diabetes alters macrophage polarization (M1/M2 imbalance), impairs neutrophil extracellular trap (NET) formation, and delays angiogenesis—all of which contribute to impaired wound healing and increased susceptibility to surgical site infection [7,8,9,10].

Consequently, diabetic patients undergoing CABG exhibit significantly higher rates of postoperative complications, including sternal wound infection, mediastinitis, prolonged mechanical ventilation, renal dysfunction, and increased reoperation rates [11]. Wound Infection (WI), particularly deep sternal wound infection (DSWI) or mediastinitis, stands out as one of the most severe and life-threatening postoperative morbidities after CABG. WI significantly prolongs intensive care and total hospital stay, increases costs, and contributes directly to both early and late mortality [12]. Evidence indicates that diabetic patients are two to four times more likely to develop WI compared to non-diabetic individuals undergoing the same procedure [13].

Further reinforcing this pattern, findings from large clinical cohorts demonstrate the disproportionate morbidity experienced by diabetic CABG patients. In an influential analysis from the CABG Patch Trial, Whang and Bigger [14] reported that diabetes was independently associated with significantly higher rates of superficial sternal wound infection and renal failure, even though long-term mortality did not significantly differ between diabetic and non-diabetic patients with severe left ventricular dysfunction. These observations underscore the substantial burden of postoperative morbidity in diabetic patients and highlight the critical role of inflammation-driven mechanisms in shaping postoperative risk.

Given the elevated vulnerability of diabetic patients to postoperative complications, identifying simple, inexpensive, and reproducible predictors that enhance preoperative risk stratification is of considerable clinical importance. In this context, inflammation-based hematologic indices have gained growing attention. Among them, the Systemic Immune–Inflammation Index (SII) has emerged as a novel composite predictor that integrates three key components of the immune–inflammatory response: platelets, neutrophils, and lymphocytes. SII, calculated as:

SII = (Platelet × Neutrophil)/Lymphocyte,

provides a multidimensional representation of the interaction between inflammation, thrombosis, and immune regulation [15].

SII has demonstrated predictive utility across various cardiovascular conditions. A recent meta-analysis by Ye, Hu [16] reported that elevated SII levels were significantly associated with increased risk of ischemic stroke, hemorrhagic stroke, myocardial infarction, and peripheral arterial disease, emphasizing its relevance to systemic vascular inflammation and cardiovascular risk [17]. Importantly, SII has shown superior prognostic performance compared with simpler indices such as the neutrophil-to-lymphocyte ratio (NLR) or platelet-to-lymphocyte ratio (PLR), as it incorporates thrombocytic, neutrophilic, and lymphocytic activity simultaneously [18].

Parallel evidence from oncologic research further reinforces the prognostic strength of SII, demonstrating significant associations between higher SII levels and poorer overall survival, greater recurrence, and more aggressive disease progression across multiple cancer types [19,20,21]. Emerging cardiovascular literature also indicates that SII correlates with the severity of coronary artery disease, plaque vulnerability, major adverse cardiovascular events (MACE), and postoperative complications following cardiac surgery [17,22,23]. Recent studies report that elevated SII predicts prolonged ICU stay, postoperative infection, and higher rates of combined morbidity after open-heart surgery [24,25]. Additionally, the glucose-to-lymphocyte ratio (GLR), another inflammation–metabolism marker, has shown predictive value for postoperative morbidity and short-term mortality in cardiovascular surgical populations [23,26].

From a mechanistic standpoint, diabetic patients are predisposed to elevated SII due to increased neutrophil activation, heightened platelet reactivity, chronic low-grade inflammation, and impaired adaptive immune responses [6]. This collection of alterations suggests that SII may be particularly informative for predicting infection-related complications such as WI in diabetic CABG patients. Furthermore, preliminary surgical studies demonstrate that perioperative increases in inflammatory indices—including SII and GLR—correlate with higher rates of postoperative infection, prolonged ventilation, and extended ICU stay [27].

Despite the growing interest in inflammation-based predictors, the predictive role of SII in diabetic patients undergoing CABG remains poorly defined. While SII has been validated in oncologic and general cardiovascular contexts, its utility for identifying high-risk diabetic patients vulnerable to postoperative WI has not been adequately examined. This knowledge gap underscores the need for studies focused specifically on the prognostic value of SII within diabetic CABG populations.

Accordingly, this study aims to evaluate the independent predictive value of preoperative SII for postoperative wound infection among diabetic patients undergoing CABG, while adjusting for established clinical, demographic, and perioperative risk factors.

## 2. Materials and Methods

### 2.1. Study Population and Design

This single-center retrospective cohort study included 300 consecutive adult patients with a confirmed diagnosis of Diabetes Mellitus (DM) who underwent isolated, elective, on-pump Coronary Artery Bypass Grafting (CABG) between January 2024 and May 2025 at a state institution in Türkiye. Patients undergoing concomitant valve surgery, emergency procedures, reoperations, or off-pump CABG were excluded. Additional exclusion criteria included lack of complete preoperative complete blood count (CBC) data, active systemic infection, chronic inflammatory or autoimmune disorders, hematologic malignancy, or ongoing immunosuppressive therapy.

Ethical approval for the study was obtained from the Institutional Ethics Committee, and the study adhered to the principles of the Declaration of Helsinki.

### 2.2. Data Collection and Definitions

Demographic characteristics, comorbid conditions, preoperative laboratory values, operative details, and postoperative outcomes were extracted from electronic medical records.

**Collected variables included**:

- **Demographics**: age, sex, and BMI.
- **Comorbidities**: hypertension (HT), kidney disease, chronic obstructive pulmonary disease (COPD), and prior myocardial infarction.
- **Laboratory parameters**: neutrophil count, lymphocyte count, and platelet count (CBC within 24 h before surgery).
- **Operative variables**: number of grafts, cardiopulmonary bypass (CPB) time, cross-clamp time, and use of left internal mammary artery (LIMA) graft.
- **Diabetes-related variables**: fasting glucose and HbA1c (when available).

### 2.3. Primary Outcome

#### 2.3.1. Wound Infection (WI)

The primary endpoint was postoperative Wound Infection (WI), defined according to the Centers for Disease Control and Prevention (CDC) and Society for Healthcare Epidemiology of America (SHEA) guidelines.

WI included:

1. Superficial sternal or harvest-site infection;
2. Deep sternal wound infection (DSWI) or mediastinitis.

Infections diagnosed during the index hospitalization or within 30 days post-discharge requiring targeted antimicrobial therapy or surgical debridement were included.

#### 2.3.2. Primary Predictor: Systemic Immune–Inflammation Index (SII)

SII was calculated using the final routine CBC obtained within 24 h preoperatively:

SII = (Platelet Count × Neutrophil Count)/Lymphocyte Count

SII was analyzed as a continuous variable. For improved interpretability, odds ratios (ORs) were reported for each 100-unit increase in SII.

### 2.4. Covariates

Multivariable logistic regression was adjusted for clinically relevant predictors of postoperative infection based on prior CABG literature:

- Age (years);
- Body Mass Index (BMI);
- Hypertension (HT);
- Kidney disease;
- Cardiopulmonary bypass duration (minutes);
- Left ventricular ejection fraction (EF) (sensitivity analyses).

In addition to the primary model, an extended multivariable model including perioperative and metabolic variables (HbA1c, operative time, transfusion, and smoking status) was constructed.

### 2.5. Statistical Analysis

Statistical analyses were performed using SPSS version 26.0.

### 2.6. Group Comparison

Continuous variables were assessed for normality using the Shapiro–Wilk test.

Normally distributed variables were compared using the Independent Samples *t*-test.

Categorical variables were compared using the Chi-square test or Fisher’s exact test when appropriate.

Data are presented as mean ± standard deviation (SD) or *n* (%).

### 2.7. Multivariable Analysis

A logistic regression model was constructed to evaluate the independent association between preoperative SII and WI.

SII: continuous variable (reported per 100-unit increase).

Results are presented as adjusted odds ratios (aOR) with 95% confidence intervals (CI).

### 2.8. Model Performance and Discrimination

The discriminative ability of SII for WI was evaluated using the receiver operating characteristic (ROC) curve.

The Area Under the Curve (AUC) and its 95% CI were calculated.

Optimal cutoff values were determined using Youden’s index.

A post-hoc power analysis was performed to evaluate the robustness of the findings. Given our sample size of 300, a wound infection incidence of 7% (*n* = 21), and the observed effect size (OR: 0.93 per 100-unit increase), the study achieved a statistical power of approximately 55%. While this is below the conventional 80% threshold, the findings should be interpreted with caution given the limited statistical power.

### 2.9. Significance Threshold

A two-sided *p*-value < 0.05 was considered statistically significant.

### 2.10. Ethical Considerations

The study protocol was approved by the institutional ethics committee, and the requirement for informed consent was waived due to the retrospective nature of the study. All procedures were conducted in accordance with the principles of the Declaration of Helsinki.

### 2.11. Declaration of Generative AI and AI-Assisted Technologies in the Writing Process

During the preparation of this work the author(s) used ChatGPT 5.1. and Gemini 1.5. Pro for language editing, clarity, and structural refinement of the manuscript. After using this tool/service, the author(s) reviewed and edited the content as needed and take(s) full responsibility for the content of the publication.

## 3. Results

### 3.1. Incidence of Wound Infection and Baseline Comparison

A total of 300 diabetic patients undergoing isolated CABG were included in the analysis. Postoperative wound infection (WI) occurred in 21 patients, corresponding to an incidence rate of 7%.

Table 1 summarizes the baseline characteristics stratified by WI status. There were no significant differences between groups regarding age, BMI, hypertension, renal disease, CRP levels, or operative times (all *p* > 0.05). However, there was a statistically significant difference in preoperative Systemic Immune–Inflammation Index (SII) values between patients with and without WI (*p* = 0.047).

Notably, and contrary to initial expectations, the mean preoperative SII was lower in patients who developed WI (958.48 ± 493.49) compared with those who did not (1293.56 ± 758.15). This inverse pattern suggests that lower systemic inflammatory activation may be associated with increased susceptibility to postoperative infection in diabetic patients.

### 3.2. Multivariable Logistic Regression Analysis

The multivariable logistic regression model evaluating predictors of postoperative WI is presented in Table 2. After adjusting for age, BMI, hypertension, and kidney disease, SII remained an independent predictor of WI (aOR = 0.93 per 100-unit increase; 95% CI: 0.86–1.00; *p* = 0.048).

Notably, while HbA1c showed a strong baseline association with WI in univariate analysis (*p* < 0.001), its independent predictive power was attenuated in the multivariable model (*p* = 0.125). This suggests that the inflammatory signal captured by preoperative SII may overlap with or partially mediate the risk associated with chronic glycemic instability.

This finding indicates that each 100-unit increase in SII was associated with a 7% reduction in the odds of WI. While counterintuitive given the traditional association between systemic inflammation and infection risk, this inverse direction may reflect diabetes-related immune dysfunction, a blunted inflammatory response, or impaired leukocyte activation, all of which may predispose patients with lower inflammatory reserve to postoperative infection.

Other covariates—including age, BMI, hypertension, and kidney disease—did not reach statistical significance.

In sensitivity analyses using SII tertiles, patients in the lowest tertile demonstrated the highest WI incidence, while the highest tertile showed the lowest risk, consistent with the primary model.

### 3.3. Comparative Analysis of Inflammatory Predictors

To further evaluate the predictive performance of SII, additional analyses were conducted comparing SII with other commonly used inflammatory indices, including the neutrophil-to-lymphocyte ratio (NLR) and platelet-to-lymphocyte ratio (PLR).

While NLR and PLR also exhibited an inverse association with postoperative wound infection, their predictive performance showed wider variance (NLR AUC: 0.419; PLR AUC: 0.237). Although all indices demonstrated poor discriminative ability, SII showed a consistent independent association with WI, unlike NLR and PLR. While all three indices showed an inverse relationship with WI, SII’s integration of three cell types may provide additional insight into the complex immune–inflammatory state of diabetic patients.

### 3.4. Discriminative Ability of SII

The ROC analysis demonstrated poor discriminative performance of SII for predicting WI, with an Area Under the Curve (AUC) of 0.374 (95% CI: 0.312–0.436; *p* = 0.047). In clinical studies, an AUC significantly below 0.50 represents an inverse predictive relationship. Therefore, this value confirms that lower preoperative SII values, rather than higher ones, are predictive of postoperative wound infection in this diabetic population (equivalent to an AUC of 0.626 when the predictor is inverted).

However, this negative discrimination aligns with the inverse association identified in the logistic regression model and further supports the observation that lower, rather than higher, SII values were associated with postoperative wound infection. The overall WI incidence was 7%, and preoperative SII values were significantly lower in patients who developed WI. These findings indicate that higher SII values may exert a protective effect, contrary to conventional expectations. The AUC value below 0.50 further confirms the inverse predictive behavior of SII. Collectively, these results support the hypothesis that immune suppression, rather than hyperinflammation, may underlie the increased risk of wound infection in diabetic CABG patients.

## 4. Discussion

This study investigated the predictive value of the preoperative Systemic Immune–Inflammation Index (SII) for postoperative wound infection (WI) in diabetic patients undergoing isolated CABG. In contrast to traditional interpretations of SII as a marker of heightened systemic inflammation and increased cardiovascular risk, we observed an inverse association between SII and WI. Higher SII values were independently associated with a reduced likelihood of WI, even after adjusting for established clinical risk factors. This unexpected finding provides novel insight into the complex interplay between systemic inflammation, immune competence, and postoperative infectious risk in diabetic surgical patients.

Furthermore, the role of SII in cardiovascular diseases extends beyond acute inflammation. Recent studies, such as Yang, Cai [28,29], have highlighted that SII and SIRI (Systemic Inflammatory Response Index) are closely associated with the severity of cerebral atherosclerosis and coronary artery lesions, especially in patients with different glucose metabolic states. While our study focused on postoperative WI, the underlying severity of systemic atherosclerosis—as reflected by SII—may contribute to impaired peripheral perfusion and subsequent wound complications.

In our comparative analysis, SII demonstrated a stronger association with postoperative wound infection than traditional inflammatory indices such as NLR and PLR. This may be attributed to its composite structure integrating neutrophil, lymphocyte, and platelet dynamics, providing a more comprehensive representation of immune–inflammatory balance.

### 4.1. A Paradoxical Relationship: Immune Competence vs. Immune Exhaustion

Diabetes Mellitus (DM) is traditionally characterized as a chronic pro-inflammatory state; however, accumulating evidence demonstrates that diabetic individuals also exhibit significant impairments in innate and adaptive immune function. Chronic hyperglycemia disrupts neutrophil chemotaxis, reduces phagocytic activity, alters macrophage polarization, and delays fibroblast-mediated tissue repair [6]. Recent immunologic research shows impaired neutrophil extracellular trap (NET) formation and dysfunctional lymphocyte signaling in diabetics [30]. Consequently, the diabetic immune system may be simultaneously “inflamed” yet functionally compromised.

From this perspective, a higher SII level—which reflects increased neutrophil and platelet activation with relative lymphocyte suppression—may represent a more functionally mobilized or primed immune state in diabetic patients, rather than pathological hyperinflammation. Conversely, lower SII values, as observed in the WI group, may reflect immune exhaustion, inadequate inflammatory reserve, or an inability to initiate a robust early immune response when confronted with surgical trauma or microbial invasion. This phenomenon has been documented in sepsis, oncology, and advanced cardiovascular disease, where immunosuppression—not inflammation—drives poor outcomes.

### 4.2. Comparison with Previous Studies

Our results differ from the majority of prior studies where higher SII values have been associated with adverse outcomes [31]. For example, Liu, Zhou [32] and Agyeman, Shafi [17] showed strong positive associations between SII and cardiovascular morbidity, including stroke, myocardial infarction, and MACE. In oncologic cohorts, elevated SII consistently predicts poor survival, advanced tumor stage, recurrence, and postoperative complications [20,33]. These findings support the classical model: higher SII = worse outcomes.

However, our findings necessitate a more nuanced interpretation in the context of diabetic surgical patients. While our results focus on the preoperative status, they arguably reflect the baseline immunological ‘reserve’ of the patient. A lower preoperative SII may indicate an underlying state of immune exhaustion or chronic metabolic stress in diabetic individuals. This baseline deficiency potentially translates into an inability to initiate a robust early immune response when confronted with subsequent surgical trauma or microbial invasion during the postoperative period. By distinguishing these temporal phases, it becomes evident that a blunted preoperative inflammatory profile may serve as a precursor to postoperative clinical vulnerability.

The discrepancy between our results and the aforementioned literature likely stems from the fact that those studies largely involved non-diabetic or mixed populations. Our findings align more closely with literature emphasizing diabetes-related immune impairment. Whang and Bigger [14] demonstrated that diabetic patients undergoing CABG experienced significantly higher morbidity—including wound complications—despite similar inflammatory marker profiles compared with non-diabetics and showed that diabetes independently increased the risk of deep sternal wound infection, independent of classical inflammatory parameters. These observations suggest that immune dysregulation, not hyperinflammation, is the primary driver of postoperative infection risk in diabetic cardiac surgery patients.

Several studies have reported that low baseline inflammation may paradoxically indicate impaired immune competence. For example, Nilsson, Liander [34] and Brattinga, Plas [35] found that patients with poor early postoperative outcomes often exhibited blunted inflammatory responses preoperatively, which may reflect underlying immunosuppression or metabolic exhaustion. In light of these findings, our results may represent a specific immunophenotype unique to diabetic CABG patients, wherein low SII denotes a compromised immune response insufficient to protect against bacterial colonization or sternal wound inflammation.

### 4.3. Biological Mechanisms Supporting the Inverse Association

Several pathophysiological mechanisms provide further support for the observed inverse relationship:

1. Impaired neutrophil function: diabetic neutrophils often exhibit reduced chemotactic accuracy and defective bactericidal capacity, regardless of absolute neutrophil count.
2. Platelet dysfunction: while diabetic patients have higher platelet reactivity, lower platelet counts (contributing to lower SII) may reflect reduced platelet-mediated immune signaling and impaired wound healing.
3. Lymphocyte dysfunction: higher lymphocyte counts (leading to lower SII) do not necessarily represent immune health; hyperglycemia impairs T-cell activation, cytokine secretion, and clonal expansion.
4. Microvascular impairment: lower systemic inflammatory activation may indicate advanced microvascular dysfunction and poorer tissue perfusion, both of which contribute to infection risk.

Together, these mechanisms may explain why diabetic patients with relatively lower preoperative SII experienced a higher risk of WI.

Our data specifically highlights a significant reduction in platelet counts within the infection group (249.83 ± 39.33 vs. 283.78 ± 45.48, *p* < 0.001). Preoperative thrombocytopenia or lower-normal platelet levels in diabetics may reflect impaired primary hemostasis and a deficiency in platelet-derived growth factors, which are essential for initiating the early phases of wound healing and local antimicrobial defense.

The prognostic superiority of composite indices over single parameters is increasingly recognized in vascular research. Recent evidence from [36] demonstrates that in patients with large-artery atherosclerosis stroke, SII exhibits significantly higher diagnostic efficacy and predictive value for disease severity compared to the Neutrophil-to-Lymphocyte Ratio (NLR). This reinforces our choice of SII as a multidimensional marker that integrates not only immune cells but also platelet activity, which is a critical driver of both thrombotic risk and wound healing impairment in diabetic CABG patients.

### 4.4. Clinical Implications

The clinical relevance of our findings is twofold. First, SII may serve as a simple, inexpensive, and widely accessible predictor that identifies diabetic CABG patients at elevated risk of postoperative WI. Second, our results call for a more nuanced interpretation of SII: in diabetes, higher SII does not necessarily indicate pathological hyperinflammation but may instead reflect preserved immune responsiveness.

Patients with low preoperative SII may benefit from:

- Intensified glycemic management;
- Optimized perioperative antibiotic strategies;
- Closer postoperative monitoring;
- Interventions targeting immune enhancement or nutritional support.

Integrating SII into current preoperative risk assessment tools may improve early recognition of vulnerable diabetic patients.

### 4.5. Limitations

This study has limitations. Its retrospective, single-center design may introduce selection bias and limit generalizability. The relatively small number of WI events reduced statistical power and restricted advanced subgroup analysis. Additionally, mechanistic immune markers (e.g., NETs, cytokine profiles, lymphocyte subtypes) were not available, limiting our ability to directly validate the immunological pathways proposed. Finally, although SII provides valuable information, it cannot replace comprehensive immunologic assessment.

### 4.6. Summary of Key Findings

In this study, SII was inversely associated with postoperative wound infection (WI), with each 100-unit increase in SII corresponding to an approximate 7% reduction in WI risk. ROC analysis further confirmed this inverse predictive pattern, demonstrating that lower SII values, rather than higher ones, were associated with increased infection risk. These findings highlight a diabetes-specific immune phenotype that may influence surgical outcomes. Accordingly, SII may serve as a clinically useful marker for identifying diabetic CABG patients at elevated risk for postoperative wound complications.

## 5. Conclusions

In conclusion, the preoperative Systemic Immune–Inflammation Index (SII) is an independent predictor of postoperative wound infection among diabetic patients undergoing CABG. However, given the modest discriminative performance observed (AUC: 0.374), SII should be interpreted with caution and ideally integrated into a broader clinical risk assessment rather than used as a standalone predictor.

Contrary to traditional patterns in non-diabetic populations, where elevated inflammation drives complications, our findings show that lower SII values are linked to increased WI risk. This inverse relationship may reflect a state of immune exhaustion or a blunted inflammatory reserve unique to the diabetic milieu. While these data underscore the importance of contextualizing inflammatory predictors, the limited number of outcome events in this cohort necessitates a conservative interpretation.

These findings should be interpreted as hypothesis-generating and require validation in larger prospective cohorts. Further large-scale, multicenter prospective studies are warranted to validate this inverse SII–WI relationship and to clarify whether enhancing the preoperative immune–inflammatory profile could mitigate infection risk in high-risk diabetic surgical populations.

## Figures and Tables

**Table 1:** Baseline Characteristics of Diabetic CABG Patients Stratified by Wound Infection Status.

Variable	No Wound Infection (*n* = 279)	Wound Infection (*n* = 21)	*p* Value
Demographics			
Age (Mean ± SD)	62.72 ± 12.92	61.24 ± 12.77	0.611
BMI (Mean ± SD)	33.63 ± 6.81	31.22 ± 7.53	0.121
Smoking, *n* (%)	74 (26.5%)	8 (38.1%)	0.231 *
Comorbidities			
Hypertension (*n* (%))	189 (67.7%)	18 (85.7%)	0.141
Kidney Disease (*n* (%))	35 (12.5%)	6 (28.6%)	0.083
Laboratory Parameters			
HbA1c (%)	7.81 ± 0.65	8.60 ± 0.87	<0.001
Neutrophils (10^3^/μL)	6.80 ± 3.52	7.02 ± 3.57	**0.782**
Lymphocytes (10^3^/μL)	1.60 ± 0.36	1.89 ± 0.42	0.001
Platelets (10^3^/μL)	283.78 ± 45.48	249.83 ± 39.33	<0.001
SII (Mean ± SD)	1293.56 ± 758.15	958.48 ± 493.49	0.047
CRP (Mean ± SD)	13.19 ± 6.51	15.86 ± 6.99	0.073
Operative Data			
CPB Time (min) (Mean ± SD)	112.54 ± 41.56	108.57 ± 36.99	0.671
Cross Clamp Time (min) (Mean ± SD)	67.29 ± 28.59	66.95 ± 31.88	0.959

Data are presented as mean ± SD or *n* (%). SII = (Platelets × Neutrophils)/Lymphocytes. Bold *p*-values indicate statistical significance (*p* < 0.05). * *p*-value calculated using Fisher’s Exact Test due to small cell frequencies (expected count < 5 in the infection group); other categorical *p*-values were derived from Pearson’s Chi-square test.

**Table 2:** Univariable and Multivariable Logistic Regression Analysis.

Variable	Univariable OR (95% CI)	*p* Value	Adjusted OR * (95% CI)	*p* Value
SII (Per 100-unit increase)	0.91 (0.84–0.99)	0.047	0.93 (0.86–1.00)	0.048
HbA1c	1.25 (1.10–1.45)	<0.001	1.18 (0.95–1.48)	0.125
Op_Time_Min	1.00 (0.99–1.01)	0.560	1.00 (0.99–1.01)	0.780
PRBC_Units	1.32 (1.00–1.75)	0.049	1.20 (0.90–1.60)	0.210
Smoker	1.45 (0.60–3.50)	0.410	1.38 (0.55–3.45)	0.490

* Due to the limited number of events (*n* = 21), the number of variables included in the multivariable model was restricted to avoid model overfitting, in accordance with recommended events-per-variable considerations.

## Data Availability

The data presented in this study are available from the corresponding author upon reasonable request. The data are not publicly available due to ethical and privacy restrictions related to patient information.

## Abbreviations

The following abbreviations are used in this manuscript:

SII	Systemic Immune–Inflammation Index
WI	Wound Infection
CABG	Coronary Artery Bypass Grafting
AUC	Area Under the Curve
